# Neonatal Intensive Care Unit Resource Use for Infants at 22 Weeks’ Gestation in the US, 2008-2021

**DOI:** 10.1001/jamanetworkopen.2024.0124

**Published:** 2024-02-21

**Authors:** Matthew A. Rysavy, Monica M. Bennett, Kaashif A. Ahmad, Ravi M. Patel, Zubin S. Shah, Dan L. Ellsbury, Reese H. Clark, Veeral N. Tolia

**Affiliations:** 1 Department of Pediatrics, McGovern Medical School at UTHealth Houston, Houston, Texas; 2 Baylor Scott & White Research Institute, Dallas, Texas; 3 The Woman’s Hospital of Texas, Houston, Texas; 4 Department of Clinical Sciences, University of Houston, Houston, Texas; 5 Pediatrix Center for Research Education, Quality, and Safety, Sunrise, Florida; 6 Department of Pediatrics, Emory University and Children’s Healthcare of Atlanta, Atlanta, Georgia; 7 Department of Pediatrics, Baylor University Medical Center, Dallas, Texas; 8 Texas A&M Health Science Center School of Medicine, Dallas, Texas; 9 MercyOne Children’s Hospital, Des Moines, Iowa

## Abstract

**Importance:**

During the past decade, clinical guidance about the provision of intensive care for infants born at 22 weeks’ gestation has changed. The impact of these changes on neonatal intensive care unit (NICU) resource utilization is unknown.

**Objective:**

To characterize recent trends in NICU resource utilization for infants born at 22 weeks’ gestation compared with other extremely preterm infants (≤28 weeks’ gestation) and other NICU-admitted infants.

**Design, Setting, and Participants:**

This is a serial cross-sectional study of 137 continuously participating NICUs in 29 US states from January 1, 2008, through December 31, 2021. Participants included infants admitted to the NICU. Data analysis was performed from October 2022 to August 2023.

**Exposures:**

Year and gestational age at birth.

**Main Outcomes and Measures:**

Measures of resource utilization included NICU admissions, NICU bed-days, and ventilator-days.

**Results:**

Of 825 112 infants admitted from 2008 to 2021, 60 944 were extremely preterm and 872 (466 [53.4%] male; 18 [2.1%] Asian; 318 [36.5%] Black non-Hispanic; 218 [25.0%] Hispanic; 232 [26.6%] White non-Hispanic; 86 [9.8%] other or unknown) were born at 22 weeks’ gestation. NICU admissions at 22 weeks’ gestation increased by 388%, from 5.7 per 1000 extremely preterm admissions in 2008 to 2009 to 27.8 per 1000 extremely preterm admissions in 2020 to 2021. The number of NICU admissions remained stable before the publication of updated clinical guidance in 2014 to 2016 and substantially increased thereafter. During the study period, bed-days for infants born at 22 weeks increased by 732%, from 2.5 per 1000 to 20.8 per 1000 extremely preterm NICU bed-days; ventilator-days increased by 946%, from 5.0 per 1000 to 52.3 per 1000 extremely preterm ventilator-days. The proportion of NICUs admitting infants born at 22 weeks increased from 22.6% to 45.3%. Increases in NICU resource utilization during the period were also observed for infants born at less than 22 and at 23 weeks but not for other gestational ages. In 2020 to 2021, infants born at less than or equal to 23 weeks’ gestation comprised 1 in 117 NICU admissions, 1 in 34 of all NICU bed-days, and 1 in 6 of all ventilator-days.

**Conclusions and Relevance:**

In this serial cross-sectional study of 137 US NICUs from 2008 to 2021, an increasing share of resources in US NICUs was allocated to infants born at 22 weeks’ gestation, corresponding with changes in national clinical guidance.

## Key Points

**Question:**

How has resource use in US neonatal intensive care units (NICUs) changed in light of updated clinical guidance about intensive care for infants born at 22 weeks’ gestation?

**Findings:**

In this serial cross-sectional study of 825 112 infants admitted to 137 NICUs in 29 US states from 2008 to 2021, there was a 388% increase in NICU admissions, 732% increase in bed-days, and 946% increase in ventilator-days for 22-week infants compared with other extremely preterm infants. Increases in resource use also occurred for infants born at less than 22 and at 23 weeks.

**Meaning:**

These findings suggest that an increasing share of resources in US NICUs is allocated to infants born at 22 weeks’ gestation.

## Teaser

This cross-sectional study examines trends in neonatal intensive care unit (NICU) resource utilization for infants born at 22 weeks’ gestation compared with other extremely preterm infants.

## Introduction

Approximately 1 in 2000 live births in the US occurs at 22 weeks’ gestation,^1^ yet infants born at 22 weeks account for 1 in 16 US infant deaths.^2^ Intensive care for infants born at 22 weeks has been controversial, with some US hospitals universally forgoing intensive care and others providing it to nearly all live-born infants.^3^ Although few published case reports of survival at 21 weeks’ gestation exist,^4^ nearly one-half of 23-week infants survived in a recent (2013-2018) US cohort.^5^ Between these extremes, approximately 30% of actively treated 22-week infants survive to hospital discharge in the US, although rates vary among hospitals.^6,7^

From 2014 to 2019, the provision of active postnatal intervention for infants born at 22 weeks’ gestation increased in the US.^8^ This change corresponded with updated clinical recommendations published during this period, including from a Eunice Kennedy Shriver National Institute of Child Health and Human Development workshop in 2014,^9^ the American College of Obstetricians and Gynecologists and Society for Maternal-Fetal Medicine in 2015,^10^ the American Academy of Pediatrics in 2015,^11^ and the Neonatal Resuscitation Program in 2016.^12^ Previous guidance from these organizations recommended forgoing intensive care for infants born at 22 weeks. The updated guidance supports its provision when desired by the family. The aim of this study was to describe resource utilization in US neonatal intensive care units (NICUs) for infants born at 22 weeks’ gestation in light of the updated guidance.

## Methods

### Population

This cross-sectional study used data from the Pediatrix Clinical Data Warehouse,^13^ a large deidentified data set with daily measures of health care utilization used by the National Institutes of Health,^14^ Food and Drug Administration,^15^ and independent investigators to describe NICU care in the US.^16,17^ The data set was created using BabySteps (Pediatrix), a clinical documentation and billing software updated daily by health care practitioners with data items specifically developed for the NICU. The use of deidentified data was approved with waiver of consent by the Western Institutional Review Board, in accordance with 45 CFR §46. This study followed the Strengthening the Reporting of Observational Studies in Epidemiology (STROBE) reporting guidelines.

The study included 137 NICUs, in 29 US states, that provided intensive care to extremely preterm infants (≤28 weeks’ gestation) and participated continuously in the data set. NICUs were located in both community and academic hospitals in urban and nonurban areas and admitted a median (IQR) of 365 (216-569) infants per year during the study, similar to a sample of US NICUs in another recent report.^18^ On the basis of a 2021 estimate of 854 level III and IV NICUs in the US, our cohort represented 16.0% of US NICUs.^19^

All NICU-admitted infants discharged between January 2008 and December 2021 from participating NICUs were included in analyses. For some analyses, NICU data were compared with publicly available data from the Centers for Disease Control and Prevention on registered US live births.^1^ We estimated that our study cohort included 16.3% of US extremely preterm infants (47 001 of 288 291 infants) for whom NICU admission was recommended for the study duration (ie, >24 weeks), a proportion consistent throughout the study (eTables 1 and 2 in Supplement 1).

### Measures of Health Care Utilization

We described health care utilization using 3 measures: NICU admissions, NICU bed-days, and ventilator-days. Admission was documented as a binary variable in the data set and represented utilization of any NICU bed. A NICU bed-day was defined as 1 calendar day during an infant’s admission. For example, an infant admitted and discharged on the same calendar day occupied 1 bed-day, and an infant discharged the following day occupied 2 bed-days. Ventilator-days were calculated in the same way as any calendar day during which an infant received mechanical ventilation through an endotracheal tube.

### Other Measures

Gestational age and other infant characteristics were assigned by the health care practitioner and used for medical care. These characteristics included birth weight, infant sex, mode of delivery, exposure to antenatal corticosteroids, whether the pregnancy was singleton or multiple, and 5-minute Apgar score. Documented maternal race and ethnicity were included to assess whether changes in care differed by these variables, as previously reported.^20,21,22^

### Statistical Analysis

Data analysis was performed from October 2022 to August 2023. For all analyses, infants were grouped by NICU and discharge years. In the primary analyses, rates of NICU admission, bed-days, and ventilator-days were standardized to rates for other extremely preterm infants (eg, per 1000 admissions of infants of ≤28 weeks’ gestational age) to account for underlying trends in preterm infant medical care that may have occurred during the 14-year period. Counts of resource use without standardization were tabulated and presented separately. Discharge years were reported in 2-year categories to minimize the number of comparisons and reduce imprecision from small numbers. To help contextualize our findings, we also described resource use for all infants of any gestational age admitted to participating NICUs and compared our findings with US population-based live births using publicly available data from birth certificates.^1^

Tests of trend were performed using Cochran-Armitage tests for binary variables, general linear model contrasts for normally distributed continuous variables, and Kendall τ correlations for ordinal and skewed variables. To determine the association of updated clinical guidance with NICU admissions, we conducted an interrupted time series analysis estimated by least-squares regression comparing the trend in admissions for the periods of 2008 to 2015 and 2016 to 2021. Analyses were performed using SAS statistical software version 9.4 (SAS Institute). Two-sided *P* values presented in each table were adjusted using the Benjamini-Hochberg method to account for multiple statistical tests. Adjusted *P *< .05 was considered statistically significant.

## Results

**Figure 1.**
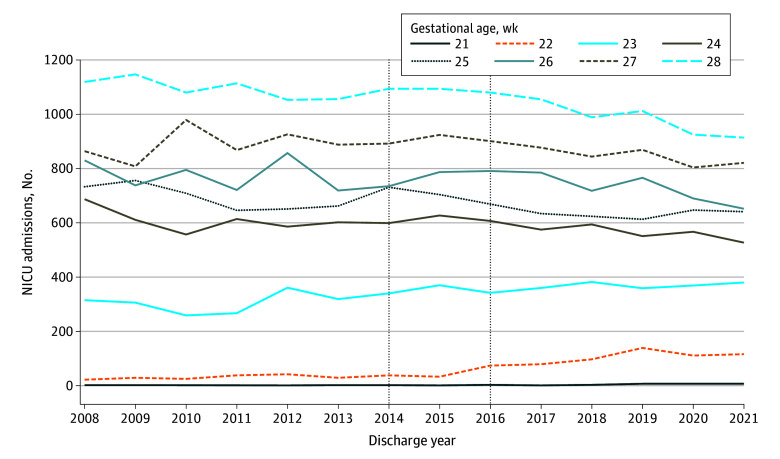
Neonatal Intensive Care Unit (NICU) Admissions by Gestational Age and Year, 2008-2021 Vertical lines represent the period (2014-2016) during which relevant US guidelines^9,10,11,12^ were published supporting the provision of intensive care for infants born at 22 weeks’ gestation when desired by the family.

Of 824 112 infants admitted to 137 NICUs from 2008 to 2021, 60 944 (7.4%) were extremely preterm and 872 (0.1%) were born at 22 weeks’ gestation (466 [53.4%] male; 18 [2.1%] Asian; 318 [36.5%] Black non-Hispanic; 218 [25.0%] Hispanic; 232 [26.6%] White non-Hispanic; 86 [9.8%] other [ie, American Indian or Alaska Native, Pacific Islander, missing, or any other race not otherwise specified] or unknown). NICU admission at 22 weeks’ gestation increased by 388%, from 5.7 per 1000 extremely preterm admissions in 2008 to 2009 to 27.8 per 1000 admissions in 2020 to 2021 (Table 1). Admissions for infants born at less than 22 and 23 weeks’ gestation also increased. Despite increasing 22-week NICU admissions, the total number of extremely preterm NICU admissions decreased by 8.8% during the study (eTable 1 in Supplement 1). Figure 1 shows the number of NICU admissions at each gestational age by year.

**Table 1.** Standardized NICU Admissions by Gestational Age and Year, 2008-2021

Gestational age, wk	No. of NICU admissions/1000 extremely pretern birth admissions							Change, %^a^	*P* value^b^
	2008-2009	2010-2011	2012-2013	2014-2015	2016-2017	2018-2019	2020-2021		
<22	0.4	0	0.3	0.3	0.5	1.2	1.7	284	<.001
22	6	7	8	8	17	28	28	388	<.001
23	69	61	78	79	80	87	92	32	<.001
24	145	135	136	137	134	134	134	−8	.07
25	166	156	150	160	148	144	158	−5	.01
26	175	175	180	170	178	173	164	−6	.11
27	186	213	207	202	201	200	199	7	.77
28	253	253	241	244	242	234	225	−11	<.001

Abbreviation: NICU, neonatal intensive care unit.

^a^ Percentage change is calculated as follows: (rate in 2020-2021 – rate in 2008-2009) / rate in 2008-2009.

^b^ *P* values were calculated by test for trend.

The interrupted time series analysis (eTable 3 in Supplement 1) showed no change in the number of admissions of 22-week infants during the study period before the updated guidance, followed by a sustained increasing trend in admissions following the publication of updated clinical guidance (increase of 8.3 admissions per year [95% CI, 1.5-15.1 admissions per year] in 2016-2021 vs 2008-2015; *P* = .04). Except for infants born at 22 weeks’ gestation and earlier, no increases in the number of admissions were noted at other gestational ages following the updated guidance. Relative changes in admissions over time are shown in the eFigure in Supplement 1.

Regarding infants born before 22 weeks’ gestation, there were 38 infants admitted to 23 NICUs during the study period. Of the 38 infants, 34 were born at 21 weeks, and 4 were born at 20 weeks; 14 were born at 21 weeks 5/7 days or 21 weeks 6/7 days. Mean (SD) birth weight was 453 (87) g. Of the 38 infants, 23 had more than 1 NICU bed-day, 6 survived to discharge, and 2 were transferred out of participating hospitals and had unknown survival outcomes. In 2020 to 2021, 11 of 137 NICUs (8.0%) admitted infants born at less than 22 weeks’ gestation.

### Changes in NICU Bed-Days

Of 15 555 075 NICU bed-days from 2008 to 2021, 4 335 391 (27.9%) were for extremely preterm infants and 39 784 (2.6%) were for infants born at 22 weeks’ gestation. NICU bed-days for infants at 22 weeks’ gestation increased by 732%, from 2.5 per 1000 extremely preterm infant bed-days in 2008 to 2009 to 20.8 per 1000 in 2020 to 2021 (Table 2). NICU bed-days for infants born at 23 weeks’ gestation also increased. Overall, the total number of extremely preterm NICU bed-days during the study changed by less than 1% (eTable 4 in Supplement 1).

**Table 2.** Standardized Neonatal Intensive Care Unit Bed-Days and Ventilator-Days by Gestational Age and Year, 2008-2021

Bed-days and ventilator-days by gestational age, wk	No. of days/1000 extremely preterm birth days							Change, %^a^	*P* value^b^
	2008-2009	2010-2011	2012-2013	2014-2015	2016-2017	2018-2019	2020-2021		
Bed-days									
<22	<0.1	0	<0.1	0.2	<0.1	0.2	0.9	2294	.13
22	3	4	6	3	12	16	21	732	.03
23	56	50	71	75	77	80	85	51	.03
24	151	145	149	147	146	146	144	−5	.13
25	193	177	165	180	165	164	173	−10	.13
26	190	191	192	179	193	189	177	−7	.45
27	185	209	203	202	198	197	199	7	.45
28	221	224	214	214	210	207	200	−10	.03
Ventilator-days									
<22	0.1	0	0.2	0.4	<0.1	0.5	1.8	1700	.10
22	5	8	13	9	26	39	52	946	.02
23	105	104	150	152	164	167	173	65	.02
24	239	249	263	262	253	246	256	7	.65
25	255	249	214	237	211	213	213	−17	.03
26	184	184	173	156	168	165	150	−18	.03
27	119	120	108	113	106	94	95	−21	.03
28	93	87	79	71	73	75	59	−36	.03

^a^ Percentage change is calculated as follows: (rate in 2020-2021 – rate in 2008-2009) / rate in 2008-2009.

^b^ *P* values were calculated by test for trend.

### Changes in Ventilator-Days

Of 1 431 135 NICU ventilator-days from 2008 to 2021, 977 133 (68.3%) were for extremely preterm infants and 20 386 (1.4%) were for infants born at 22 weeks’ gestation. Ventilator-days for infants born at 22 weeks’ gestation increased by 946%, from 5.0 per 1000 extremely preterm infant ventilator-days in 2008 to 2009 to 52.3 per 1000 in 2020 to 2021 (Table 2). Ventilator-days for infants born at 23 weeks’ gestation also increased. Overall, the total number of extremely preterm infant ventilator-days decreased by 24.1% during the study (eTable 5 in Supplement 1).

### Hospital Variation

From 2008 to 2021, 104 of 137 NICUs (75.9%) admitted infants born at 22 weeks. This compares to 23 (16.8%) that admitted infants born at less than 22 weeks and 133 (97.1%) that admitted 23-week infants. NICUs admitting 22-week infants increased from 31 of 137 (22.6%) in 2008 to 2009 to 62 (45.3%) in 2020 to 2021 (eTable 6 in Supplement 1). NICUs admitting infants born at less than 22 weeks also increased during the study.

The mean number of 22-week infants in admitting NICUs increased from 1.7 per hospital in 2008 to 2009 to 3.7 per hospital in 2020 to 2021 (eTable 7 in Supplement 1). There were no significant increases in the number of admitted infants per admitting NICU at other extremely preterm gestations.

### Infant Characteristics

During the 14-year study period, there were no significant changes in gestational age at birth (in days) or birth weight of 22-week infants admitted to the NICU (Table 3). There was an increase in the proportion exposed to antenatal corticosteroids (from 22 of 51 infants [43.1%] to 166 of 277 infants [73.1%]) and a decrease in the proportion of infants who were multiples (from 18 of 51 infants [35.3%] to 41 of 227 infants [18.1%]). NICU-admitted 22-week infants who died before discharge at participating hospitals decreased from 74.5% (38 of 51 infants) to 53.7% (122 of 227 infants) during the study, and survival to discharge increased from 15.7% (8 of 51 infants) to 28.2% (64 of 227 infants). The remaining infants were transferred out of participating hospitals at a median (IQR) age of 34 (6-91) days and had unknown survival outcomes. Among infants who died, the median (IQR) age at death increased from 1 (0-8) to 3 (0-11) days.

**Table 3.** Characteristics of Infants Born at 22 Weeks’ Gestation, 2008-2021

Characteristics	Infants, No. (%)							*P* value
	2008-2009 (n = 51)	2010-2011 (n = 63)	2012-2013 (n = 71)	2014-2015 (n = 71)	2016-2017 (n = 153)	2018-2019 (n = 236)	2020-2021 (n = 227)	
Gestational age at birth								
22 wk 0-3 d	31 (60.8)	37 (58.7)	34 (47.9)	34 (47.9)	79 (51.6)	107 (45.3)	113 (49.8)	.09
22 wk 4-6 d	20 (39.2)	26 (41.3)	37 (52.1)	37 (52.1)	74 (48.4)	129 (54.7)	114 (50.2)	
Birth weight, mean (SD), g	516.6 (79.9)	505.3 (67.4)	505.5 (92.4)	522.1 (81.1)	508.8 (79.3)	512.9 (80.8)	520.6 (188.3)	.65
Birth weight <400 g	2 (3.9)	2 (3.2)	8 (11.3)	5 (7.0)	9 (5.9)	14 (5.9)	21 (9.3)	.27
Sex								
Male	30 (58.8)	37 (58.7)	39 (54.9)	39 (54.9)	74 (48.4)	133 (56.4)	114 (50.2)	.23
Female	21 (41.2)	26 (41.3)	32 (45.1)	32 (45.1)	79 (51.6)	103 (43.6)	113 (49.8)	
Multiple birth	18 (35.3)	16 (25.4)	20 (28.2)	13 (18.3)	39 (25.5)	49 (20.8)	41 (18.1)	.01
Race and ethnicity								
Asian	3 (5.9)	1 (1.6)	0	0	3 (2.0)	5 (2.1)	6 (2.6)	<.001
Black Non-Hispanic	12 (23.5)	17 (27.0)	29 (40.8)	27 (38.0)	56 (36.6)	95 (40.3)	82 (36.1)	
Hispanic	12 (23.5)	21 (33.3)	19 (26.8)	22 (31.0)	38 (24.8)	56 (23.7)	50 (22.0)	
White Non-Hispanic	16 (31.4)	23 (36.5)	18 (25.4)	14 (19.7)	42 (27.5)	60 (25.4)	59 (26.0)	
Other or unknown^a^	8 (15.7)	1 (1.6)	5 (7.0)	8 (11.3)	14 (9.2)	20 (8.5)	30 (13.2)	
Antenatal steroids	22 (43.1)	33 (52.4)	29 (40.8)	25 (35.2)	87 (56.9)	137 (58.1)	166 (73.1)	<.001
Cesarean delivery	23 (45.1)	27 (42.9)	17 (23.9)	21 (29.6)	48 (31.4)	73 (30.9)	67 (29.5)	.06
Outborn^b^	10 (19.6)	10 (15.9)	11 (15.5)	19 (26.8)	21 (13.7)	34 (14.4)	34 (15.0)	.25
5-min Apgar score, median (IQR)	5 (3-6)	4 (2-7)	5 (3-6)	5 (2-6)	4 (2-7)	4 (2-6)	4 (2-6)	.11
Outcomes								
Transferred^c^	4 (7.8)	3 (4.8)	7 (9.9)	8 (11.3)	29 (19.0)	35 (14.8)	41 (18.1)	<.01
Mortality	38 (74.5)	47 (74.6)	49 (69)	51 (71.8)	89 (58.2)	148 (62.7)	122 (53.7)	<.001
Survival to discharge	8 (15.7)	13 (20.6)	15 (21.1)	12 (16.9)	35 (22.9)	53 (22.5)	64 (28.2)	.03
Age at death among those who died, median (IQR), d	1 (0-8)	1 (0-8)	1 (0-2)	2 (0-4)	1 (1-7)	2 (0-9)	3 (0-11)	.01

^a^ Other includes American Indian or Alaska Native, Pacific Islander, missing, or any other race not otherwise specified.

^b^ Refers to infants born outside of hospitals included in study.

^c^ Refers to infants who were transferred to hospital outside of this study before discharge home.

### Comparison With All NICU Admissions

In 2008 to 2009, infants of 23 weeks’ gestation or less comprised 0.6% (1 in 164) of all NICU admissions, 1.7% (1 in 59) of all NICU bed-days, and 7.3% (1 in 14) of all ventilator-days. In 2020 to 2021, infants of 23 weeks’ gestation or less comprised 0.9% (1 in 117) of NICU admissions, 2.9% (1 in 34) of all NICU bed-days, and 16.0% (1 in 6) of all ventilator-days (eTables 1, 4, and 5 in Supplement 1).

**Figure 2.**
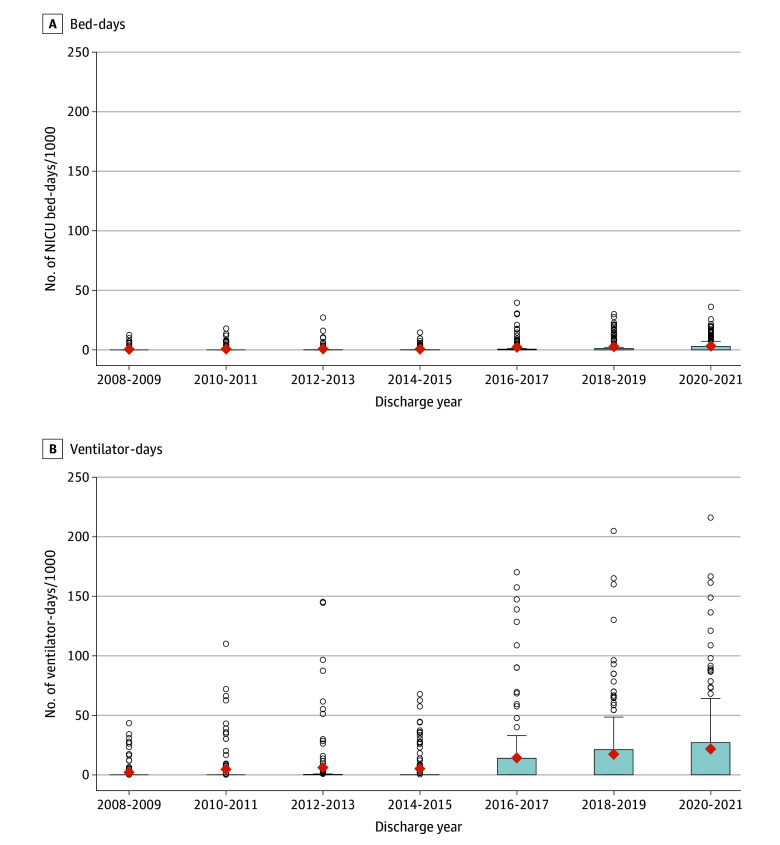
Proportion of All Neonatal Intensive Care Unit (NICU)–Admitted Infant Bed-Days and Ventilator-Days for Infants Born at 22 Weeks’ Gestation by Year and Admitting NICU, 2008-2021 Data shown are from all NICU admissions regardless of gestational age. Box-and-whisker plots show the 25th and 75th percentiles (boxes), mean (diamond), and 90th percentile (error bars). Outlier hospitals are shown as circles. The median for all years for both measures was 0.

Figure 2 shows the proportions of all NICU bed-days and ventilator-days utilized by 22-week infants at each NICU. In 2020 to 2021, 22-week infants utilized greater than 50 per 1000 ventilator-days at 23 (16.8%) NICUs and greater than 100 per 1000 ventilator-days in 7 (5.1%) NICUs.

### US Live Birth Trends

The changes observed in this cohort of US NICUs occurred in the context of decreasing extremely preterm live births in the US between 2008 and 2021 (eTable 2 in Supplement 1).^1^ US live births at 22 weeks’ gestation decreased from 4534 in 2008 to 2009 to 3268 in 2020 to 2021, a 27.9% decrease. Live births at 21 to 28 weeks’ gestation decreased by 20.4% between 2008 to 2009 and 2020 to 2021, consistent with the observed decrease in NICU admissions of infants born at 28 weeks’ gestation or less in our data set during the same period (eTable 1 in Supplement 1).

## Discussion

This cross-sectional study found that, from 2008 to 2021, NICU resource utilization for infants born at 22 weeks’ gestation increased substantially, as shown by a 388% increase in NICU admissions, 732% increase in bed-days, and 946% increase in ventilator-days compared with other extremely preterm infants. These changes occurred following the publication of updated clinical guidance regarding intensive care at 22 weeks’ gestation. Substantial increases in NICU resource utilization were also observed for infants born at less than 22 and at 23 weeks’ gestation but not other extremely preterm infants. Notably, these changes occurred in the context of fewer infants born at less than or equal to 23 weeks’ gestation in the US.

Our findings are consistent with other recent publications showing increasing treatment and survival at 23 weeks’ gestation or less in the US. In a cohort of US hospitals participating the Vermont-Oxford Network, active postnatal treatment at 22 weeks increased from 26% of liveborn infants in 2008 to 58% in 2019, and survival increased from 6% to 17%, with sustained increases in treatment beginning in 2015.^8^ The findings are also similar to the observation of increased active treatment at 22 weeks’ gestation in the UK following publication of updated clinical guidance by the British Association of Perinatal Medicine.^23^

Our study indicates that increased NICU resource utilization for 22-week infants in the US was related to increasing numbers of NICUs providing intensive care for 22-week infants, increasing admissions in these hospitals, and increasing infant survival. The nearly 10-fold increase in the proportion of extremely preterm ventilator-days utilized by 22-week infants appears to be related to both decreasing use of mechanical ventilation for infants born at 25 to 28 weeks and the extended duration of mechanical ventilation in survivors born at 22 weeks.^17,24^

Notably, we observed increasing provision of intensive care for infants born at less than 22 weeks’ gestation in the US, with 8% of NICUs (11 of 137 NICUs) admitting infants at less than 22 weeks in 2020 to 2021. Current clinical guidance does not recommend intensive care for infants born before 22 weeks’ gestation.^9,10,12^

### Strengths and Limitations

Strengths of this study include the use of a data set with relevant daily measures of resource utilization in a diverse cohort of US NICUs and the ability to place the care of extremely preterm infants in the context of other NICU-admitted infants from 2008 to 2021.^25^ Notably, the changes observed in this study preceded the 2022 Supreme Court decision in *Dobbs v Jackson Women’s Health Organization*, which affected laws regarding pregnancy termination across the US, with potential implications for both trends in live births and NICU admissions of extremely preterm infants. Our findings should be of value to clinicians and policymakers to understand the impact of increasing intensive care for infants born at 23 weeks’ gestation or earlier during the past decade on resource allocation and the interpretation of metrics related to NICU processes and outcomes.^26^

Our study also has several important limitations. First, data used to measure resource utilization were hospital based, not population based. Second, although the NICUs included are diverse in geographic distribution and patient demographics, they represent only 1 in 6 US level III and IV NICUs. Third, our study did not describe resource utilization for infants not admitted to the NICU, such as antenatal counseling or the presence of a neonatologist at delivery, but included data for admitted infants only. Fourth*,* our data set did not contain information on health care expenditures, an important measure of resource utilization. An analysis of infants born from 2008 to 2016 enrolled with a single US insurer estimated 6-month health care expenditures of $603 778 (2016 USD) per infant born at 24 weeks’ gestation and $242 886 per infant born at less than or equal to 23 weeks.^27^ These estimates preceded the substantial increases in admission, survival, and bed-day and ventilator-day utilization observed in our analyses. Updated estimates of health care expenditures for infants born at less than or equal to 23 weeks are needed. Fifth*,* we were unable to account for medical care or outcomes before admission or following transfer from participating NICUs. Data from this report are relevant to understanding patterns of NICU utilization but should not be used for determining prognosis or for antenatal counseling for individual infants.

## Conclusions

In this serial cross-sectional study of 137 US NICUs from 2008 to 2021, NICU resource utilization for infants born at 22 weeks’ gestation substantially increased following publication of updated clinical guidance. Increases in NICU resource utilization were also seen for infants at less than 22 and at 23 weeks’ gestation but not other extremely preterm infants.

## Notes

Supplement 1. **eTable 1.** Number of NICU Admissions by Gestational Age and Year, 2008-2021 **eTable 2.** U.S. Livebirths by Gestational Age and Year, 2008-2021 **eTable 3.** Association of Updated Clinical Guidance and NICU Admissions by Gestational Age and Year, 2008-2021 **eTable 4.** NICU Bed-Days by Gestational Age and Year, 2008-2021 **eTable 5.** Ventilator-Days by Gestational Age and Year, 2008-2021 **eTable 6.** Number of Admitting Hospitals by Gestational Age and Year, 2008-2021 **eTable 7.** Mean Admissions per Hospital by Gestational Age and Year, 2008-2021 **eFigure.** Relative Change in Standardized NICU Admissions by Gestational Age Over Time

Supplement 2.
Data Sharing Statement
