# Supplementary material for: Neonatal Intensive Care Unit Resource Use for Infants at 22 Weeks’ Gestation in the US, 2008-2021
Source: JAMA Netw Open. 2024 Feb 21;7(2):e240124. doi: 10.1001/jamanetworkopen.2024.0124 (PMC10882422; doi:10.1001/jamanetworkopen.2024.0124)
Supplement: Supplement 1. — eTable 1. Number of NICU Admissions by Gestational Age and Year, 2008-2021 eTable 2. U.S. Livebirths by Gestational Age and Year, 2008-2021 eTable 3. Association of Updated Clinical Guidance and NICU Admissions by Gestational Age and Year, 2008-2021 eTable 4. NICU Bed-Days by Gestational Age and Year, 2008-2021 eTable 5. Ventilator-Days by Gestational Age and Year, 2008-2021 eTable 6. Number of Admitting Hospitals by Gestational Age and Year, 2008-2021 eTable 7. Mean Admissions per Hospital by Gestational Age and Year, 2008-2021 eFigure. Relative Change in Standardized NICU Admissions by Gestational Age Over Time [file jamanetwopen-e240124-s001.pdf]

Supplemental Online Content

Rysavy MA, Bennett MM, Ahmad KA, et al. Neonatal intensive care unit resource use for infants at 22 weeks' gestation in the US, 2008-2021. *JAMA Netw Open*. 2024;7(2):e240124. doi:10.1001/jamanetworkopen.2024.0124

eTable 1. Number of NICU Admissions by Gestational Age and Year, 2008-2021
eTable 2. U.S. Livebirths by Gestational Age and Year, 2008-2021
eTable 3. Association of Updated Clinical Guidance and NICU Admissions by Gestational Age and Year, 2008-2021
eTable 4. NICU Bed-Days by Gestational Age and Year, 2008-2021
eTable 5. Ventilator-Days by Gestational Age and Year, 2008-2021
eTable 6. Number of Admitting Hospitals by Gestational Age and Year, 2008-2021
eTable 7. Mean Admissions per Hospital by Gestational Age and Year, 2008-2021
eFigure. Relative Change in Standardized NICU Admissions by Gestational Age Over Time

This supplemental material has been provided by the authors to give readers additional information about their work.

© 2024 Rysavy MA et al. *JAMA Network Open.*

eTable 1. Number of NICU Admissions by Gestational Age and Year, 2008-2021

	NICU Admissions, No.							
	**2008-2009**	**2010-2011**	**2012-2013**	**2014-2015**	**2016-2017**	**2018-2019**	**2020-2021**	**% Change***
**<22 weeks**	4	0	3	3	4	10	14	**250%**
**22 weeks**	51	63	71	71	153	236	227	**345%**
**23 weeks**	621	526	680	710	702	741	749	**21%**
**24 weeks**	1298	1171	1188	1226	1182	1145	1094	**-16%**
**25 weeks**	1489	1355	1313	1435	1303	1237	1288	**-13%**
**26 weeks**	1568	1516	1576	1522	1576	1484	1342	**-14%**
**27 weeks**	1672	1847	1814	1816	1778	1713	1625	**-3%**
**28 weeks**	2266	2194	2109	2188	2135	2001	1839	**-19%**
**≤28 weeks**	**8969**	**8672**	**8754**	**8971**	**8833**	**8567**	**8178**	**-9%**
**All infants**	**110,841**	**113,783**	**115,612**	**123,448**	**123,577**	**122,331**	**115,520**	**4%**

* % Change calculated as ([count in 2020-2021] – [count in 2008-2009]) / (count in 2008-2009)

© 2024 Rysavy MA et al. *JAMA Network Open.*

eTable 2. U.S. Livebirths by Gestational Age and Year, 2008-2021

	Liveborn Infants, No.							
	**2008-2009**	**2010-2011**	**2012-2013**	**2014-2015**	**2016-2017**	**2018-2019**	**2020-2021**	**% Change***
**21 weeks**	3481	3192	3204	3192	2957	2727	2407	**-31%**
**22 weeks**	4534	4242	4218	3966	3678	3584	3268	**-28%**
**23 weeks**	6117	5790	5569	5791	5708	5251	5081	**-17%**
**24 weeks**	8521	7843	7860	7537	7399	6908	6554	**-23%**
**25 weeks**	9098	8587	8490	8437	8011	7586	7231	**-21%**
**26 weeks**	10096	9695	9623	9450	9189	8592	8222	**-19%**
**27 weeks**	11758	11114	10916	10725	10509	10197	9612	**-18%**
**28 weeks**	14362	13465	12973	13180	12871	12543	11759	**-18%**
**≤28 weeks**	**67967**	**63928**	**62853**	**62278**	**60322**	**57388**	**54134**	**-20%**
**All infants**	**8,378,359**	**7,952,976**	**7,885,022**	**7,966,573**	**7,801,375**	**7,539,252**	**7,277,939**	**-13%**

Gestational age was determined by the best obstetric estimate as recorded on the birth certificate.

* % Change calculated as ([count in 2020-2021] – [count in 2008-2009]) / (count in 2008-2009)

© 2024 Rysavy MA et al. *JAMA Network Open.*

eTable 3. Association of Updated Clinical Guidance and NICU Admissions by Gestational Age and Year, 2008-2021

	Pre-guidelines trend (2008-2015)		Post-guidelines trend (2016-2021)		Change in trend	
**Gestational age**	**Beta (95% CI)**	**p-value**	**Beta (95% CI)**	**p-value**	**Beta (95% CI)**	**p-value**
<22 weeks	-0.1 (-0.5, 0.3)	0.66	1.2 (0.6, 1.8)	0.003	1.3 (0.6, 2.0)	0.005
22 weeks	1.6 (-2.0, 5.3)	0.40	9.9 (4.2, 15.6)	0.007	8.3 (1.5, 15.1)	0.04
23 weeks	9.9 (1.4, 18.3)	0.05	5.5 (-7.5, 18.6)	0.43	-4.3 (-19.9, 11.2)	0.60
24 weeks	-4.4 (-14.0, 5.1)	0.39	-13.3 (-28.2, 1.5)	0.11	-8.9 (-26.6, 8.8)	0.35
25 weeks	-5.5 (-16.2, 5.2)	0.34	-3.2 (-19.7, 13.3)	0.71	2.3 (-17.4, 22.0)	0.82
26 weeks	-4.9 (-18.9, 9.2)	0.51	-26.6 (-48.4, -4.8)	0.04	-21.8 (-47.7, 4.2)	0.13
27 weeks	7.4 (-5.2, 20.1)	0.28	-17.0 (-36.5, 2.6)	0.12	-24.4 (-47.7, -1.1)	0.07
28 weeks	-6.8 (-15, 1.4)	0.13	-34.2 (-46.9, -21.5)	<0.001	-27.4 (-42.5, -12.3)	0.005

Interrupted time series analysis to evaluate the association between updated U.S. clinical guidance and NICU admissions. Clinical guidance prior to 2014-2016 recommended against the provision of intensive care for infants born at 22 weeks' gestation, whereas the published updates supported the provision of intensive care for infants born at 22 weeks' gestation when desired by the family. Beta coefficient values represent the annual change in the number of babies treated at each week of gestation during each period. The "change in trend" value represents a comparison of the baseline trends.

© 2024 Rysavy MA et al. *JAMA Network Open.*

eTable 4. NICU Bed-Days by Gestational Age and Year, 2008-2021

	NICU Bed Days, No.							
	**2008-2009**	**2010-2011**	**2012-2013**	**2014-2015**	**2016-2017**	**2018-2019**	**2020-2021**	**% Change***
**<22 weeks**	24	0	27	130	5	132	569	**2271%**
**22 weeks**	1,491	2,437	3,425	2,196	7,398	10,336	12,501	**738%**
**23 weeks**	34,042	30,015	43,816	48,522	48,983	50,409	50,915	**50%**
**24 weeks**	91,724	86,621	91,806	94,335	92,576	92,737	86,415	**-6%**
**25 weeks**	117,438	106,275	101,504	115,576	104,787	104,218	104,145	**-11%**
**26 weeks**	115,475	114,625	117,949	115,419	122,449	119,944	106,429	**-8%**
**27 weeks**	112,620	125,066	124,442	129,712	125,844	124,838	119,838	**6%**
**28 weeks**	134,478	134,336	131,416	137,738	133,567	131,138	120,538	**-10%**
**≤28 weeks**	**607,292**	**599,375**	**614,385**	**643,628**	**635,609**	**633,752**	**601,350**	**-1%**
**All infants**	**2,114,281**	**2,154,956**	**2,206,029**	**2,301,556**	**2,298,568**	**2,284,251**	**2,195,434**	**4%**

* % Change calculated as ([count in 2020-2021] – [count in 2008-2009]) / (count in 2008-2009)

© 2024 Rysavy MA et al. *JAMA Network Open.*

eTable 5. Ventilator-Days by Gestational Age and Year, 2008-2021

	Ventilator-Days, No.							
	**2008-2009**	**2010-2011**	**2012-2013**	**2014-2015**	**2016-2017**	**2018-2019**	**2020-2021**	**% Change***
**<22 weeks**	24	0	26	54	3	63	224	**833%**
**22 weeks**	842	1,194	1,796	1,225	3,461	5,203	6,665	**692%**
**23 weeks**	17,564	15,154	20,586	20,477	21,516	22,147	22,029	**25%**
**24 weeks**	40,070	36,222	36,218	35,477	33,161	32,521	32,591	**-19%**
**25 weeks**	42,791	36,170	29,414	32,020	27,698	28,222	27,131	**-37%**
**26 weeks**	30,844	26,851	23,736	21,103	22,026	21,870	19,153	**-38%**
**27 weeks**	19,991	17,403	14,899	15,235	13,937	12,381	12,041	**-40%**
**28 weeks**	15,626	12,587	10,899	9,602	9,525	9,897	7,568	**-52%**
**≤28 weeks**	**167,752**	**145,581**	**137,574**	**135,193**	**131,327**	**132,304**	**127,402**	**-24%**
**All infants**	**251,397**	**214,815**	**202,772**	**198,489**	**193,501**	**189,877**	**180,284**	**-28%**

* % Change calculated as ([count in 2020-2021] – [count in 2008-2009]) / (count in 2008-2009)

© 2024 Rysavy MA et al. *JAMA Network Open.*

eTable 6. Number of Admitting Hospitals by Gestational Age and Year, 2008-2021

	2008-2009	2010-2011	2012-2013	2014-2015	2016-2017	2018-2019	2020-2021	% Change*	p-value**
**<22 weeks**	3 (2.2%)	0 (0.0%)	3 (2.2%)	3 (2.2%)	4 (3.0%)	9 (6.6%)	11 (8.0%)	**267%**	<0.01
**22 weeks**	31 (22.6%)	30 (21.9%)	36 (26.3%)	35 (25.5%)	56 (40.9%)	56 (40.9%)	62 (45.3%)	**100%**	<0.001
**23 weeks**	99 (72.3%)	105 (76.6%)	111 (81.0%)	109 (79.6%)	111 (81.0%)	102 (74.5%)	106 (77.4%)	**7%**	0.86
**24 weeks**	116 (84.7%)	117 (85.4%)	112 (81.8%)	122 (89.1%)	117 (85.4%)	113 (82.5%)	118 (86.1%)	**2%**	0.89
**25 weeks**	120 (87.6%)	122 (89.1%)	120 (87.6%)	120 (87.6%)	118 (86.1%)	114 (83.2%)	121 (88.3%)	**1%**	0.86
**26 weeks**	117 (85.4%)	118 (86.1%)	125 (91.2%)	123 (89.8%)	122 (89.1%)	121 (88.3%)	118 (86.1%)	**-1%**	0.86
**27 weeks**	119 (86.9%)	126 (92.0%)	129 (94.2%)	125 (91.2%)	124 (90.5%)	120 (87.6%)	122 (89.1%)	**-3%**	0.86
**28 weeks**	129 (94.2%)	131 (95.6%)	131 (95.6%)	130 (94.9%)	131 (95.6%)	127 (92.7%)	128 (93.4%)	**-1%**	0.86

* % Change calculated as ([count in 2020-2021] – [count in 2008-2009]) / (count in 2008-2009)
** Test for trend

© 2024 Rysavy MA et al. *JAMA Network Open.*

eTable 7. Mean Admissions per Hospital by Gestational Age and Year, 2008-2021

	2008-2009	2010-2011	2012-2013	2014-2015	2016-2017	2018-2019	2020-2021	% Change*	p-value**
**<22 weeks**	1.3	0	1	1	1	1.1	1.3	**-5%**	0.61
**22 weeks**	1.7	2.1	2	2	2.7	4.2	3.7	**123%**	<0.001
**23 weeks**	6.3	5	6.1	6.5	6.3	7.3	7.1	**13%**	0.13
**24 weeks**	11.2	10	10.6	10.1	10.1	10.1	9.3	**-17%**	0.42
**25 weeks**	12.4	11.1	10.9	12	11	10.9	10.6	**-14%**	0.55
**26 weeks**	13.4	12.9	12.6	12.4	12.9	12.3	11.4	**-15%**	0.36
**27 weeks**	14.1	14.7	14.1	14.5	14.3	14.3	13.3	**-5%**	0.61
**28 weeks**	17.6	16.8	16.1	16.8	16.3	15.8	14.4	**-18%**	0.29

* % Change calculated as ([rate in 2020-2021] – [rate in 2008-2009]) / (rate in 2008-2009)
** Test for trend

© 2024 Rysavy MA et al. *JAMA Network Open.*

eFigure. Relative Change in Standardized NICU Admissions by Gestational Age Over Time

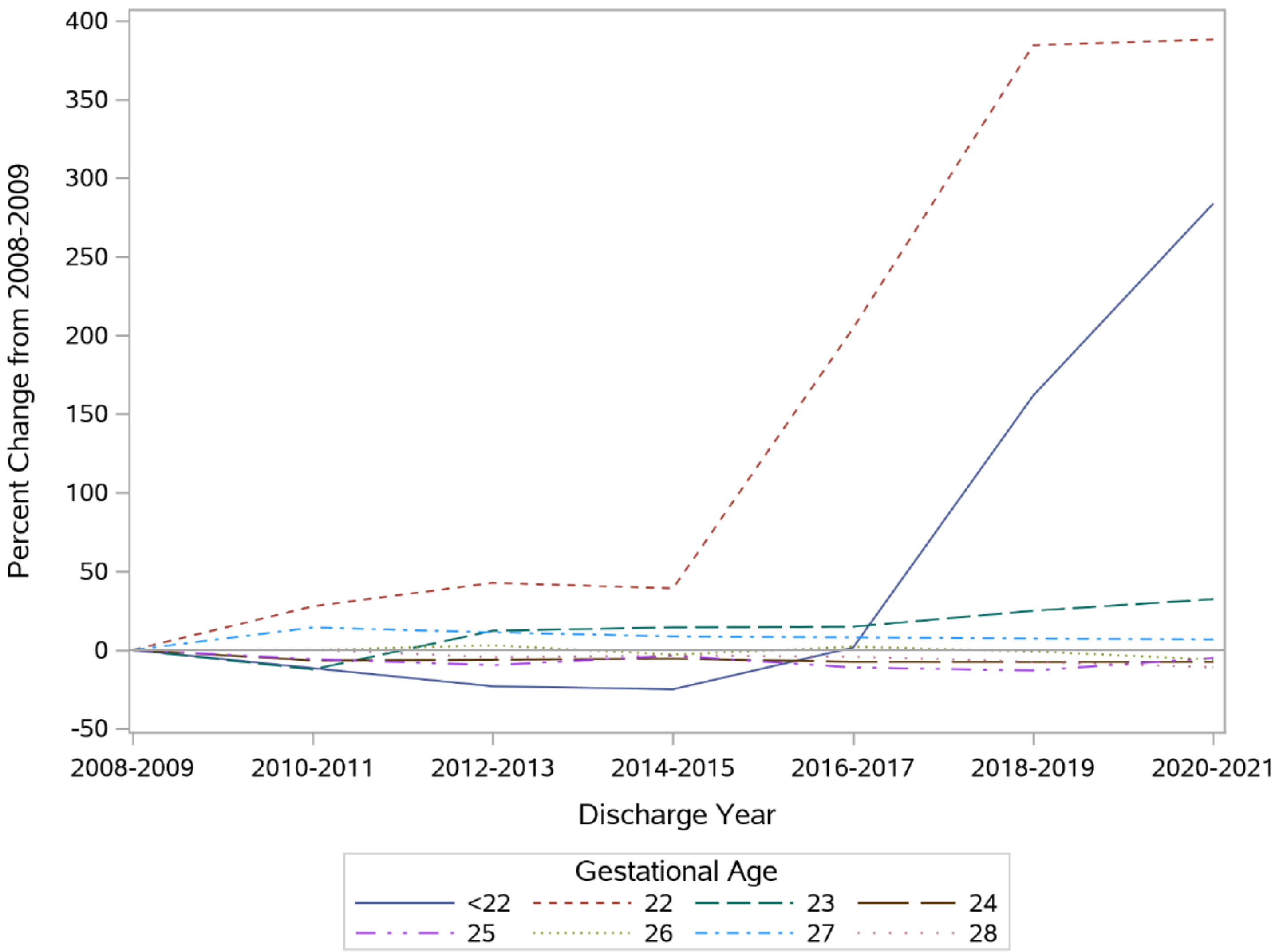

© 2024 Rysavy MA et al. *JAMA Network Open.*
