# Supplementary material for: Neonatal Intensive Care Unit Resource Use for Infants at 22 Weeks’ Gestation in the US, 2008-2021
Source: JAMA Netw Open. 2024 Feb 21;7(2):e240124. doi: 10.1001/jamanetworkopen.2024.0124 (PMC10882422; doi:10.1001/jamanetworkopen.2024.0124)
Supplement: Supplement 2. — Data Sharing Statement [file jamanetwopen-e240124-s002.pdf]

Data Sharing Statement

Rysavy. Neonatal Intensive Care Unit Resource Use for Infants at 22 Weeks' Gestation in the US, 2008-2021. *JAMA Netw Open*. Published February 21, 2024.
doi:10.1001/jamanetworkopen.2024.0124

Data
Data available: No
